# Evaluation of Pyrolysis Oil as Carbon Source for Fungal Fermentation

**DOI:** 10.3389/fmicb.2016.02059

**Published:** 2016-12-22

**Authors:** Stefan Dörsam, Jennifer Kirchhoff, Michael Bigalke, Nicolaus Dahmen, Christoph Syldatk, Katrin Ochsenreither

**Affiliations:** ^1^Technical Biology, Institute of Process Engineering in Life Sciences, Karlsruhe Institute of Technology (KIT)Karlsruhe, Germany; ^2^Thermochemical Conversation of Biomass, Institute of Catalysis Research and Technology, Karlsruhe Institute of Technology (KIT)Karlsruhe, Germany

**Keywords:** pyrolysis oil, fermentation, *Aspergillus oryzae*, *Rhizopus delemar* organic acids, carbon source, fungi, tolerance mechanisms

## Abstract

Pyrolysis oil, a complex mixture of several organic compounds, produced during flash pyrolysis of organic lignocellulosic material was evaluated for its suitability as alternative carbon source for fungal growth and fermentation processes. Therefore several fungi from all phyla were screened for their tolerance toward pyrolysis oil. Additionally *Aspergillus oryzae* and *Rhizopus delemar*, both established organic acid producers, were chosen as model organisms to investigate the suitability of pyrolysis oil as carbon source in fungal production processes. It was observed that *A. oryzae* tolerates pyrolysis oil concentrations between 1 and 2% depending on growth phase or stationary production phase, respectively. To investigate possible reasons for the low tolerance level, eleven substances from pyrolysis oil including aldehydes, organic acids, small organic compounds and phenolic substances were selected and maximum concentrations still allowing growth and organic acid production were determined. Furthermore, effects of substances to malic acid production were analyzed and compounds were categorized regarding their properties in three groups of toxicity. To validate the results, further tests were also performed with *R. delemar*. For the first time it could be shown that small amounts of phenolic substances are beneficial for organic acid production and *A. oryzae* might be able to degrade isoeugenol. Regarding pyrolysis oil toxicity, 2-cyclopenten-1-on was identified as the most toxic compound for filamentous fungi; a substance never described for anti-fungal or any other toxic properties before and possibly responsible for the low fungal tolerance levels toward pyrolysis oil.

## Introduction

To date, the production of most chemicals is still based on fossil resources, namely coal, gas, and crude oil. However, in times of climate change and declining fossil resources, the use of renewable resources and environmental friendly methods for a sustainable production of chemicals, materials and also of energy are becoming increasingly important. The basis of a bio-based economy are biotechnological processes which enable the transformation of renewable resources to value-added products. One of the biggest challenges for the establishment of such a bio-based economy is, however, the naturally insufficient utilization of lignocellulosic materials in a wide range of microorganisms. Therefore, many pretreatment methods have been developed to enable the accessibility of lignocellulosic materials in microbial processes. In the organosolv process, for instance, the main components of lignocellulose are separated and cellulose and hemicellulose fractions are subsequently saccharified. Lignin, however, remains largely inaccessible. A possible pretreatment to utilize lignocellulosic material completely by breaking all polymeric bonds, is pyrolysis resulting in pyrolysis oil. Pyrolysis oil is obtained by fast pyrolysis of wood or other lignocellulosic biomass. It offers a substrate, which can be generated from nearly every dry biomass, not competing with food or feed and which is much more independent from season and region than other biomass-based substrates for fermentation. The three key components of lignocellulose (lignin, cellulose, and hemicellulose) are decomposed and depolymerized to monomeric sugars, small aromatic compounds and further low-molecular substances (Oasmaa and Czernik, 1999) making pyrolysis oil a possible resource for fermentative processes (Neumann et al., 2015). The main components are organic acids, sugars, hydroxyaldehydes, hydroxyketones, and phenolic compounds. Their proportion depends largely on the process parameters like residence time, pressure, temperature, and heating rate. Some ingredients are promising as substrate for microorganisms. Microbial utilization of pyrolysis oil depends highly on detoxification and fractionation. It was shown that acetic acid is a possible carbon source for malic acid production with *Aspergillus oryzae* (Oswald et al., 2016). Other organisms, e.g., *Cupriavidus necator*, were able to use acetic acid and propanoic acid as carbon sources for growth and the production of polyhydroxyalkanoates (PHA), particularly polyhydroxybutyrate (PHB). Pyrolytic sugars, mostly levoglucosan (1,6-anhydro-β-D-glucopyranose) have the very best biotechnological potential of the pyrolysis fractions. However, most organisms are not able to metabolize levoglucosan. Therefore, the intern ether bond of levoglucosan can be hydrolyzed by acidic treatment and thus, levoglucosan is converted to glucose. The acid-treated sugar fraction can then be used for fermentation, e.g., ethanol production with *Saccharomyces cerevisiae* (Chan and Duff, 2010; Lian et al., 2010). On the other hand, direct application of levoglucosan without pretreatment is possible when working with naturally levoglucosan utilizing microorganisms. As shown by Prosen et al. (1993) several yeasts are able to grow on detoxified pyrolysis oil and also fungi of the genera *Aspergillus* and *Penicillium* can convert levoglucosan directly to glucose-6-phosphate which is the first intermediate of the glycolysis. This and the known robustness, makes fungi the most promising organisms for pyrolysis oil utilization. The aim of this study is to evaluate the suitability of crude pyrolysis oil as a carbon source for fungal growth and fungal fermentation processes. Therefore a variety of fungi from all phyla were tested for their tolerance to crude pyrolysis oil. Tolerance and toxicity tests with representative model substances in several concentrations, were analyzed for their effect on growth and their effects of malic acid production of *A. oryzae* and fumaric acid production with *R. delemar* was studied.

## Materials and Methods

### Chemicals

All chemicals, including selected substances from pyrolysis oil were either purchased from Sigma–Aldrich (Munich, Germany) or Roth (Karlsruhe, Germany).

### Preparation of Pyrolysis Oil

The pyrolysis oil used in this study was prepared from wheat straw by fast pyrolysis in the bioliq plant at KIT (bioliq^®^). This process has been developed to convert biomass into a liquid fuel for various applications to produce heat, electricity, and transportations fuels. Small and dry biomass particles of a few mm size are rapidly heated up by a heat carrier (e.g., sand) in a pneumatically or mechanically fluidized bed at 500 ± 30°C in the absence of oxygen. This process is described by Henrich et al. (2016). In case of pyrolysis oils obtained from herbaceous biomass, higher water amounts are formed, which can cause phase separation of the pyrolysis oil. A phase rich in organic compounds (which was used in this study) and an aqueous phase consisting of up to 80 wt. Percentage of water and water soluble organic compounds are formed.

The analyzed monomeric substances are compiled as determined by Thünen Institute Hamburg by GC-MS in the section “Supplementary Material.”

### Fungi and Media

The fungal strains used, *A. oryzae* DSM 1863 and *R. delemar* DSM 905, were obtained from the DSMZ strain collection (Deutsche Sammlung von Mikroorganismen und Zellkulturen, Braunschweig, Germany). *A. oryzae* was grown on minimal medium (MM) for *Aspergillus* spec. (Barratt et al., 1965): 6 g/L NaNO_3_, 0.52 g/L KCl, 0.52 g/L MgSO_4_⋅7H_2_O, and 1.52 g/L KH_2_PO_4_. The pH was set to 6.5 with NaOH. 10 g/L glucose, 2 mL of 1000x Hutner’s Trace Elements, and 15 g/L agar were added after autoclaving. 1000 x Hutner’s Trace Element solution consists of 5 g/L FeSO_4_⋅7H_2_O, 50 g/L EDTA-Na_2_, 22 g/L ZnSO_4_⋅7H_2_O, 11 g/L H_3_BO_3_, 5 g/L MnCl_2_⋅4H_2_O, 1.6 g/L CoCl_2_⋅6H_2_O, 1.6 g/L CuSO_4_⋅5H_2_O, and 1.1 g/L (NH_4_)_6_Mo_7_O_24_⋅4H_2_O, pH 6.5 (Barratt et al., 1965). *R. delemar* was grown on modified supplemented agar (SUP) (modified from Wöstemeyer, 1985): 10 g/L glucose, 0.5 g/L yeast extract, 4 g/L KH_2_PO_4_, 0.9 g/L K_2_HPO_4_, 4 g/L NH_4_Cl, 0.25 g/L MgSO_4_⋅7H_2_O. The pH was set to 6.5 with NaOH.

For conidia collection, *A. oryzae* was grown on high-salt minimal medium (Song et al., 2001) which contains additionally 22.37 g/L KCl. For spore collection, *R. delemar* was grown on malt extract agar (MEA): 30 g/L malt extract, 3 g/L peptone, 15 g/L agar. The conidia and spores were harvested with 50% glycerol from plates that were incubated for 5 days at 30°C and filtered through Miracloth (Calbiochem). The spore/conidia solution was diluted to a concentration of 1 × 10^7^ (spore/conidia)/mL and stored at -80°C.

Fungi for pyrolysis oil tolerance tests were either obtained from DSMZ, ATCC (American Type Culture Collection), NRRL (Northern Regional Research Laboratory) or JMRC (Jena Microbial Resource Collection) and grown on MM (*Alternaria alternata* DSM 12633*, Aspergillus niger* NRRL 3, *Aspergillus terreus* DSM 5770*, Aspergillus nidulans* DSM 820*, Penicillium chrysogenum* ATCC 48271), yeast minimal medium (YMM) (*Aureobasidium pullulans* DSM 2404*, Candida bombicola* ATCC 22214*, Saccharomyces cerevisiae* DSM 11285*, Yarrowia lipolytica* DSM 1345*, Cryptococcus curvatus* ATCC 20508*, Phanerochaete chrysosporium* DSM 1547*, Pleurotus ostreatus* DSM 11191*, Trametes versicolor* DSM 3086*, Mucor circinelloides* SF 006299) or modified SUP (*Backusella circina* SF 000941*, Mortierella elongata* SF 009721*, Phycomyces blakesleeanus* SF 018907*, Rhizopus microspores* STH 00427*, Umbelopsis ramanniana* SF 011341). YMM contains 20 g/L glucose and 6.7 g/L yeast nitrogen base. All media were sterilized by autoclaving.

Organic acid production was accomplished in a two-step process with a pre-culture and a main culture. The pre-culture medium for *A. oryzae* consists of 40 g/L glucose monohydrate, 4 g/L (NH_4_)_2_SO_4_, 0.75 g/L KH_2_PO_4_, 0.98 g/L K_2_HPO_4_⋅3H_2_O, 0.1 g/L MgSO_4_⋅7H_2_O, 0.1 g/L CaCl_2_⋅2H_2_O, 5 mg/L NaCl, and 5 mg/L FeSO_4_⋅7H_2_O. Main culture medium for *A. oryzae* contains 120 g/L glucose monohydrate, 1.2 g/L (NH_4_)_2_SO_4_, 0.1 g/L KH_2_PO_4_, 0.17 g/L K_2_HPO_4_⋅3H_2_O, 0.1 g/L MgSO_4_⋅7H_2_O, 0.1 g/L CaCl_2_⋅2H_2_O, 5 mg/L NaCl, and 60 mg/L FeSO_4_⋅7H_2_O.

The pre-culture medium for *R. delemar* consists of 30 g/L glucose, 2.0 g/L urea, 0.6 g/L KH_2_PO_4_, 0.5 g/L MgSO_4_⋅7H_2_O, 0.11 g/L ZnSO_4_, 8.8 mg/L FeSO_4_⋅7H_2_O. The pH was set to 4.5 with 10 M HCl after autoclaving to support growth in form of pellets. Main culture medium for *R. delemar* consists of 100 g/L glucose, 0.2 g/L urea, 0.6 g/L KH_2_PO_4_, 0.5 g/L MgSO_4_⋅7H_2_O, 0.11 g/L ZnSO_4_, 8.8 mg/L FeSO_4_⋅7H_2_O. The media were sterilized by autoclaving. To keep the pH above 5.5 during fermentation, 90 g/L CaCO_3_ was added to both main culture media. For inhibition experiments main culture medium was mixed with the indicated amount of chemicals or pyrolysis oil.

### Germination and Growth Inhibition Analysis

To prepare testing plates, different concentrations of the respective substances were added to the agar containing MM for *A. oryzae* or modified SUP for *R. delemar* directly after autoclaving. To determine the inhibitory concentration of pyrolysis derived substances on growth and germination, agar plates were inoculated onto the middle of the plate with 4 × 10^4^ conidia/spores. After incubation for 3 days at 30°C the diameter of the colony was determined every day over 5 days using a ruler. For the inhibitory concentration of pyrolysis oil, conidia/spores were streaked onto MM/SUP agar plates with different amounts of pyrolysis oil. For pyrolysis oil tolerance tests with all other fungi, spores, conidia or mycelium fragments were transferred on agar plates containing pyrolysis oil and incubated for 5 days.

To promote agar solidification after addition of pyrolysis oil, the pH was set to 6 by titration with NaOH.

### Organic Acid Production

For *A. oryzae* pre-culture, 100 mL of pre-culture medium was filled into 500 mL Erlenmeyer shake flasks and inoculated with 2 × 10^7^ conidia. The flasks were incubated at 100 rpm and 30°C for 24 h in a rotary shaker. To remove the pre-culture medium, fungal pellets were washed twice with distilled water. 100 mL of main culture was transferred to 500 mL Erlenmeyer shake flasks and mixed with 9 g/L sterile CaCO_3_. The flasks were inoculated with 10% (*v*/*v*) of washed pre-culture and incubated at 120 rpm and 32°C for 7 days.

For *R. delemar* pre-culture, 100 mL of pre-culture medium was filled into 500 mL Erlenmeyer shake flasks and inoculated with 1 × 10^7^ spores. The flasks were incubated at 100 rpm and 35°C for 30 h in a rotary shaker. To remove the pre-culture medium, fungal pellets were washed twice with distilled water. 100 mL of main culture was transferred to 500 mL Erlenmeyer shake flasks and mixed with 9 g/L sterile CaCO_3_. The flasks were inoculated with 10% (*v*/*v*) of washed pre-culture and incubated at 120 rpm and 35°C for 7 days.

For both fungi, the first sample was taken after 72 h and subsequently every 48 h.

### Organic Acid Analytics

For malic and fumaric acid quantification with HPLC, fermentation broth samples were pretreated and analyzed as described in Ochsenreither et al. (2014) with minor modifications. To re-dissolve the precipitated calcium malate/fumarate, 1 mL of well-mixed sample was mixed with 1 mL of 3 M H_2_SO_4_ and 3 mL of distilled water and incubated at 80°C for 20 min. 1 mL of the mixture was transferred to a 1.5 mL Eppendorf tube and centrifuged in a table top centrifuge for 5 min at 20,000 × *g*. The supernatant was used for HPLC analysis, which was performed with a standard HPLC device (Agilent 1100 Series, Agilent, Germany) prepared with a 15 cm reversed phase column (Synergi^TM^4 μm Fusion-RP 80 Å, LC Column 150 × 4.6 mm, Phenomenex, Aschaffenburg, Germany) at 30°C. Mobile phase solution A was 100% methanol, and solution B was 20 mM KH_2_PO_4_, pH 2.5. The flow rate was 1 mL/min and a gradient was used for the separation of organic acids: 0–0.5 min 100% eluent B, 0.5–10-min increase of eluent A from 0 to 10%, 10–12-min a further increase of eluent A from 10 to 70%, 12–14 min a decrease of eluent A from 70 back to 0%, and 14–18 min again 100% eluent B. The increase of eluent A to 70% from 10 to 12 min was applied to elute and analyze the tested hydrophobic substances which were added to the medium. The injection volume was 10 μL and the detection was performed by a UV detector at 220 nm. Standards were used for peak identification and calibration. The linear detection range went from 0.1 to 5 g/L malic acid and 0.02 to 0.5 g/L fumaric acid.

## Results

### Pyrolysis Oil as Carbon Source for Fungi

To determine the pyrolysis oil tolerance limits of fungi, fungal species of all phyla (Ascomycota, Basidiomycota, and Zygomycota) were either streaked out, or mycelium fragments were transferred on agar plates containing in addition to glucose different concentrations of pyrolysis oil. The results are summarized in **Table 1**.

**Table 1:** Growth of fungi on minimal agar plates depending on addition of different concentrations of pyrolysis oil from 0 to 3%.

Phylum	Organism	Pyrolysis oil content (% w/v)^a^
		
		0	0.5	1	2	3
*Ascomycota*	*Alternaria alternata*	+	+	-	-	-
	*Aspergillus niger*	+	+	-	-	-
	*Aspergillus terreus*	+	+	+	-	-
	*Aspergillus nidulans*	+	+	-	-	-
	*Aspergillus oryzae*	+	+	+	+	-
	*Aureobasidium pullulans*	+	+	+	-	-
	*Candida bombicola*	+	+	+	-	-
	*Penicillium chrysogenum*	+	-	-	-	-
	*Saccharomyces cerevisiae*	+	+	+	-	-
	*Yarrowia lipolytica*	+	+	-	-	-
	*Trigonopsis variabilis*	+	-	-	-	-
*Basidiomycota*	*Cryptococcus curvatus*	+	-	-	-	-
	*Phanerochaete chrysosporium*	+	+	+	-	-
	*Pleurotus ostreatus*	+	-	-	-	-
	*Trametes versicolor*	+	+	+	+	+
*Zygomycota*^b^	*Backusella circina*	+	+	-	-	-
	*Mortierella elongata*	+	-	-	-	-
	*Mucor circinelloides*	+	+	+	-	-
	*Phycomyces blakesleeanus*	+	+	-	-	-
	*Rhizopus microsporus*	+	-	-	-	-
	*Rhizopus delemar*	+	+	+	-	-
	*Umbelopsis ramanniana*	+	-	-	-	-

^a^*Media contain 10 g/L glucose*.

^b^*Former phylum contains: Entomophthoromycotina, Kickxellomycotina, Mucoromycotina, Zoopagomycotina.*

Most of the analyzed fungi tolerated a pyrolysis oil content of 0.5 %. Only *P. chrysogenum, T. variabilis, C. curvatus, P. ostreatus, M. elongata, R. microspores*, and *U. ramanniana* were not able to grow under these conditions. 1% is above the upper tolerance limit of *A. alternata, A. niger*, *A. nidulans*, *Y. lipolytica*, *B. circina*, and *P. blakesleeanus*. Higher concentrations were only tolerated by *A. oryzae*, which grew up to a pyrolysis oil content of 2% and *T. versicolor* which grew on all tested concentrations. Additionally colonies of *T. versicolor* showed a dark/black halo on pyrolysis oil containing agar plates.

The results showed a great range of tolerance across the kingdom of fungi. Beside tolerance, the metabolization of pyrolysis oil is necessary for it to be used as carbon source for fungal fermentation. Because of the ability to produce a value added product and the highest tolerance limit for pyrolysis oil, further tests were conducted with *Aspergillus oryzae* and additionally with *Rhizopus delemar*.

*A. oryzae* was able to grow on sugar free medium containing up to 1% of pyrolysis oil as sole carbon source. Therefore, *A. oryzae* is able to metabolize substances within pyrolysis oil for biomass production. However, on plates containing more than 1% of pyrolysis oil as sole carbon source, growth was not observed (data not shown). Due to the fact that organic acid production takes place during the stationary growth phase, the fermentation process is substantially different to active growth. Therefore, the effect of pyrolysis oil on malic acid production has to be investigated separately. Consequently, malic acid production was tested in the presence of 0–3% pyrolysis oil. By using pyrolysis oil as sole carbon source, malic acid production was not observed.

**FIGURE 1:**
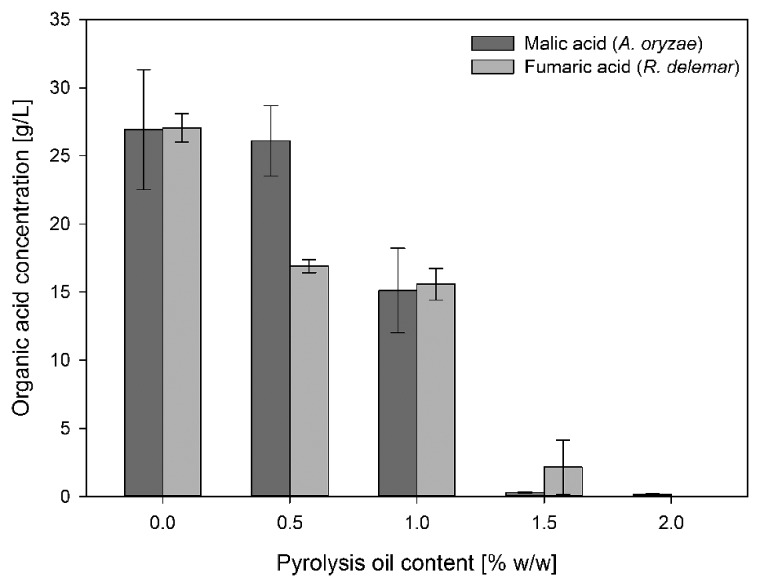
**Influence of different pyrolysis oil concentrations in production medium on malic acid production by *A. oryzae* DSM 1863 and fumaric acid production by *R. delemar* DSM 905**. The experiments were done in shake flasks cultivated at 120 rpm and 32/35°C. All concentrations are given as average of three independent experiments ± standard deviation.

The comparison between tolerance tests (medium contains glucose and pyrolysis oil in various concentrations) and utilization tests (medium contains only pyrolysis oil in various concentrations) indicates that pyrolysis oil can be tolerated in higher concentrations by fungi when glucose is the main carbon source. However, with increasing pyrolysis oil content fungal growth is more and more restrained and the production of malic acid and fumaric acid by *A. oryzae* and *R. delemar*, respectively, is strongly reduced even in the presence of glucose (**Figure 1**).

With respect to malic acid production by *A. oryzae*, the addition of 0.5% of pyrolysis oil to organic acid production medium has no influence compared to medium without pyrolysis oil (26.09 ± 2.59 g/L to 26.92 ± 4.40 g/L), whereas fumaric acid production by *R. delemar* is strongly influenced in the presence of 0.5% of pyrolysis oil (27.04 ± 1.04 g/L to 16.90 ± 0.43 g/L). Higher concentrations led to a successive decrease of malic acid production with *A. oryzae* from 15.12 ± 3.11 g/L at 1%, to finally 0.3 ± 0.03 g/L and 0.18 ± 0.03 g/L with 1.5 and 2% pyrolysis oil content, respectively. Between 0.5 and 1% there was only a small decrease in fumaric acid production with *R. delemar* to 15.58 ± 1.18 g/L. Higher concentration led also to a successive decrease of final organic acid concentration to 2.15 ± 1.98 g/L at 1.5% and 0 with 2%.

### Toxicity Analysis of Pyrolysis Oil Derived Substances

Results of the tolerance and utilization tests showed that crude pyrolysis oil is in principle suitable as carbon source for fungi; however, applicable concentrations are too low for most processes and for organic acid production in particular. The elucidation of substances problematic for growth and organic acid production is therefore a prerequisite for further application of pyrolysis oil in biotechnology. By avoiding the formation of the identified substances during fast pyrolysis by adjusting process parameters of by decreasing their content below the critical concentration by fractionation might be a practical solution. For this, 11 representative and commercially available substances which are present in higher concentrations in the oil, were selected and tested for the more tolerant *A. oryzae*. A selection of these were also tested for *R. delemar*. The chosen concentrations were based on the amount found in pyrolysis oil as the upper limit. Some of the analyzed substances showed no inhibition effects, whereas some had a great impact even in low concentrations. An overview of the analyzed chemicals and their inhibitory concentrations for *A. oryzae* are shown in **Table 2** and for *R. delemar* in **Table 3**.

**Table 2:** Overview of growth and malic acid production limits of *A. oryzae* depending on different concentrations of pyrolysis oil derived substances.

Tested substances	Concentration in pyrolysis oil (% w/w)	Growth limit (% w/w)^a^	Malic acid production limit (% w/w)^a^
Propionic acid	1.302	0.07	>1.3
Ethylene glycol	1.258	>1.25	>1.25
γ-Butyrolactone	0.335	>0.335	>0.335
Hydroxyacetone	4.4631	1.5	2.5
Syringol^∗^	0.556	0.27	0.3
Guaiacol	0.469	0.1	0.1
Furfural	0.281	0.03	0.07
Phenol	0.384	0.07	0.07
Isoeugenol^∗^	0.524	0.03	0.06
*o*-,-*m*,-*p*-Cresol	0.17	0.05	0.03
2-Cyclopenten-1-on	0.308	0.00625	0.0125

*^a^Values represent the highest tested concentrations where *A. oryzae* was still able to grow/produce malic acid. In the next higher tested concentration growth/production was not observed. ^∗^Analysis indicates a degradation of substances during cultivation. ‘>’ Limit is above highest tested concentration*.

**Table 3:** Overview of growth and fumaric acid production limits of *R. delemar* depending on different concentrations of pyrolysis oil derived substances.

Tested substances	Concentration in pyrolysis oil (% w/w)	Growth limit (% w/w)^a^	Fumaric acid production limit (% w/w)^a^
Propionic acid	1.302	0.1	>1.3
Hydroxyacetone	4.4631	1.5	1.5
Isoeugenol	0.524	0.025	0.005
2-Cyclopenten-1-on	0.308	0.005	0.005

*^a^Values represent the highest tested concentrations where *R. delemar* was still able to grow/produce fumaric acid. In the next higher tested concentration growth/production was not observed. ‘>’ Limit is above highest tested concentration*.

### Growth Limits

When observing the influence of the selected compounds on the growth behavior of *A. oryzae* compared to control, the obtained results could be divided in two groups. The first group comprises of substances with very low influence to the growth of *A. oryzae* in the analyzed concentrations. This group contains ethylene glycol and γ-butyrolactone. It was concluded that the maximum tolerance levels are probably much higher than the concentrations in pyrolysis oil, so that these substances will not be accounted as problematic. The second group contains all other analyzed substances. These substances showed a considerable inhibition to fungal growth when added in concentrations relevant to their content in pyrolysis oil. Typically, growth is reduced even at the lowest tested concentration when compared to the control. For *R. delemar* all substances in the tested concentration could be classified to the second group.

In the growth experiments with *A. oryzae*, agar medium containing syringol, a yellow/orange colored substance, was decolorized around the fungal colony indicating for degradation or derivatization of syringol. As indicated in **Table 2**, the analysis of the substances gave in some cases hints for their degradation.

### Organic Acid Production Limit

Several substances from pyrolysis oil were tested for their effects on the organic acid production of *A. oryzae* and *R. delemar* and their inhibition limits were detected. The analyzed concentrations and the resulting yields in relation to the respective organic acids are shown in **Tables 4** and **5**.

**Table 4:** Overview of tested substances from pyrolysis oil and tested concentrations and their effects to malic acid production yields of *A. oryzae*.

Tested substances	Concentrations in main culture medium (%)	*Y*_P/S_ (g/g)
Control (10% Glucose)		0.64
Propionic acid	1.3	0.66
	1	0.65
	0.5	0.67
	0.4	0.69
	0.3	0.74
	0.1	0.59
	0.07	0.58
	0.05	0.61
Hydroxyacetone	2.5	0.42
	2	0.37
	1.5	0.39
	1	0.54
Isoeugenol	0.06	0.35
	0.05	0.60
2-Cyclopenten-1-on	0.0125	0.40
	0.00625	0.38
	0.003125	0.46
Ethylene glycol	1.25	0.56
	1.2	0.48
	1	0.62
	0.7	0.51
	0.5	0.65
	0.3	0.66
γ-Butyrolactone	0.335	0.59
	0.3	0.57
	0.25	0.55
	0.2	0.56
	0.15	0.52
	0.1	0.49
Syringol	0.3	0.04
	0.27	0.06
	0.25	0.06
	0.23	0.09
	0.2	0.17
	0.17	0.20
Guaiacol	0.1	0.82
	0.07	0.69
	0.005	0.65
Furfural	0.07	0.67
	0.05	0.54
	0.03	0.55
	0.02	0.56
	0.01	0.57
Phenol	0.07	0.25
	0.05	0.52
	0.03	0.86
*o*-,-*m*,-*p*-Cresol	0.03	0.53
	0.02	0.62
	0.01	0.74

*Tested substance concentrations with no observed production are not shown*.

**Table 5:** Overview of tested substances from pyrolysis oil and tested concentrations and their effects to fumaric acid production yields of *R. delemar*.

Tested substances	Concentration in main culture medium (%)	*Y*_P/S_ (g/g)
Control (10% Glucose)		0.38
Propionic acid	1.3	0.51
	1	0.10
	0.7	0.22
	0.5	0.21
	0.25	0.26
Hydroxyacetone	1.5	0.15
Isoeugenol	0.005	0.02
2-Cyclopenten-1-on	0.005	0.10

*Tested substance concentrations with no observed production are not shown*.

**FIGURE 2:**
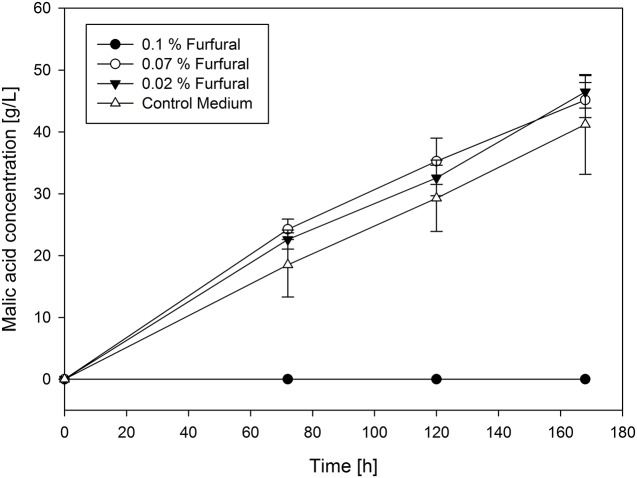
**Selected production curves of malic acid depending on different furfural concentration added to the main culture medium**. As reference main culture medium without furfural was used. Shake flasks were incubated at 32°C for 7 days. Samples were taken every 48 h. All values are given as average of minimum three independent experiments ± standard deviation.

In contrast with the growth inhibition experiments, malic acid production is affected in a more complex way by the added substances. In the control approach, a yield of malic acid production with *A. oryzae* of 0.64 g/g could be achieved. Based on the production curves appearance, the chemicals tested can be divided into three groups. The first one contains propionic acid, cresol, ethylene glycol, 2-cyclopenten-1-on, furfural, guaiacol and γ-butyrolactone. Regarding γ-butyrolactone, ethylene glycol and propionic acid all tested concentrations showed no influence on malic acid production compared to their absence. This is also valid for the yields, with a range from 0.48 g/g to 0.66 g/g with ethylene glycol and 0.49 g/g to 0.59 g/g with γ-butyrolactone. 0.3% of propionic acid in the medium even led to a substantially higher yield for malic acid production (0.74 g/g). For furfural (0.54–0.67 g/g), guaiacol, cresol and 2-cyclopenten-1-one (0.38–0.46 g/g) no inhibition of malic acid production was observed until a certain critical concentration of substances was added. In fact, malic acid production was even promoted in the presence of cresol and guaiacol compared to the control approach, also with higher yields (up to 0.82 g/g with guaiacol and 0.74 g/g with cresol) until the critical concentration was reached. However, the transition from no influence to total inhibition of production is very abrupt. Selected production curves of furfural are shown in **Figure 2** as representative of this group.

The produced concentration of malic acid after 7 days of fermentation with furfural addition ranged between 43.59 ± 2.69 g/L and 46.48 ± 2.63 g/L, with ethylene glycol addition between 36.04 ± 3.26 g/L and 44.59 ± 2.36 g/L, with propionic acid addition between 33.66 ± 2.47 g/L and 44.41 ± 4.29 g/L. With 2-cyclopenten-1-on addition the concentration ranged between 32.91 ± 7.45 g/L and 39.05 ± 9.2 g/L and with cresol addition between 40.09 ± 2.37 g/L and 67.07 ± 14.49 g/L. For guaiacol addition the produced final malic acid concentration ranged between 43.66 ± 27.09 and 61.45 ± 20.91. The control approach resulted in a malic acid concentration of 41.21 ± 8.06 g/L. Except 2-cyclopenten-1-on, substances from this group are therefore considered as moderately problematic in concentrations relevant to their content in pyrolysis oil but have also been shown to promote malic acid production below a certain threshold.

**FIGURE 3:**
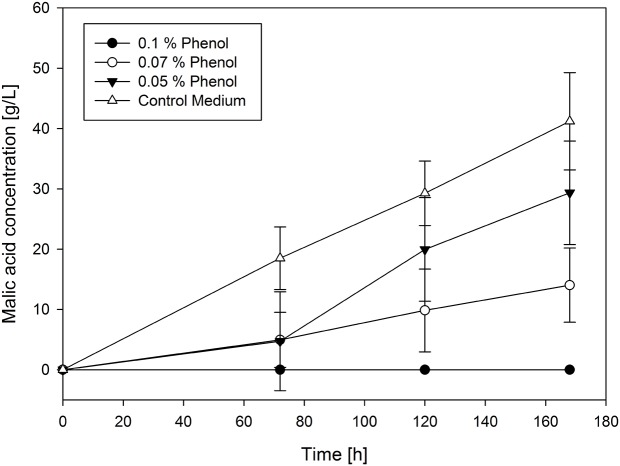
**Selected production curves of malic acid depending on different phenol concentration added to the main culture medium**. As reference main culture medium without phenol was used. Shake flasks were incubated at 32°C for 7 days. Samples were taken every 48 h. All values are given as average of minimum three independent experiments ± standard deviation.

The second group includes most of the remaining substances. Regarding hydroxyacetone, phenol and syringol, malic acid production correlates directly with their concentration in the main culture medium. For these substances the transition from no influence to total inhibition is smooth. As an example of this group a selection of the production curves of phenol is shown in **Figure 3**.

It was observed that lower concentrations of phenol increased malic acid production when compared to the absence of phenol. By adding 0.03% of phenol, malic acid concentration raised to 53.79 ± 1.25 g/L after 168 h compared to the control approach with 41.21 ± 8.06 g/L. Above this limit concentration of phenol decreased the production. This is also valid for the yields, where 0.03% of phenol leads to the highest yield of 0.86 g/g. In contrast, higher concentrations of phenol decreased the yield until 0.25 g/g with 0.07%. Similarly, the addition of 1% hydroxyacetone led to the production of 46.19 ± 8.09 g/L malic acid which is slightly higher than in the control approach but with a lower yield (0.54 g/g). Lower concentrations of added hydroxyacetone had no influence on malic acid production, whereas higher concentrations of hydroxyacetone decreased the production. Yields ranged from 0.37 g/g to 0.54 g/g in tested concentrations. However, the lowest tested concentration (0.17%) of syringol resulted in a much lower concentration of malic acid (21.83 ± 5.13 g/L) than in the control approach. The resulting yields for all syringol concentrations were in a very long range, between 0.04 g/g (with 0.3%) and 0.2 g/g (with 0.17%), increasing the yield with the decreasing syringol concentration.

**FIGURE 4:**
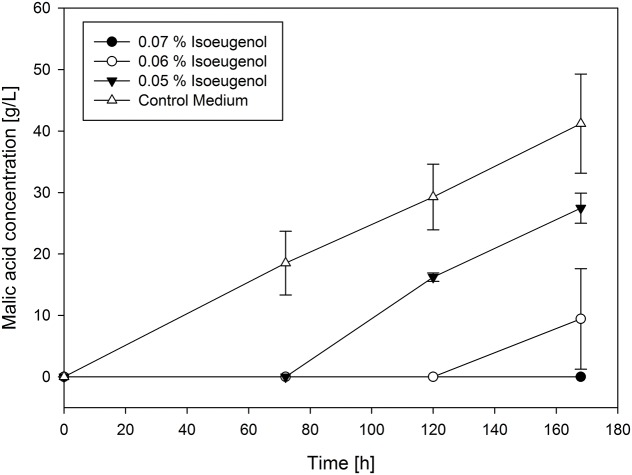
**Selected production curves of malic acid depending on different isoeugenol concentration added to the main culture medium**. As reference main culture medium without isoeugenol was used. Shake flasks were incubated at 32°C for 7 days. Samples were taken every 48 h. All values are given as average of minimum three independent experiments ± standard deviation.

The last group of malic acid production curves contains only one member. Isoeugenol showed considerable evidence of degradation by the fungus during the fermentation process. A selection of the production curves of isoeugenol are shown in **Figure 4**.

For all tested concentrations, malic acid production was delayed depending on initial isoeugenol concentration. At a concentration of 0.07% production was not observed during cultivation. The lowest tested isoeugenol concentration (0.05%) resulted in much lower malic acid production (27.46 ± 2.45 g/L) than in the control approach, but with a very similar yield of 0.6 g/g. This was lower for 0.6% isoeugenol (0.35 g/g), which could be a hint for degradation of isoeugenol during fermentation (**Table 2**).

The categorization into several groups of fumaric acid production inhibition of *R. delemar* is not possible because of the small amount of tested substances and larger concentration intervals. The yields were in general very low compared to the control approach with 0.38 g/g, except the yield for 1.3% propionic acid, which is higher than the control (**Table 3**). By decreasing the propionic acid concentration to 0.25%, fumaric acid yield increased to 0.26 g/g. Only one single, very low concentration could be found for hydroxyacetone (0.15 g/g), 2-cyclopenten-1-on (0.1 g/g) and isoeugenol (0.02 g/g) where product formation could be observed.

## Discussion

Pyrolysis oil as a complex mixture of organic compounds is an interesting but challenging substrate for fermentation. Besides sugars and organic acids, which are easily assessable as carbon sources, many substances are present which have not been studied for their influence on microorganisms and which might be problematic for growth or production. So far, the main focus of biotechnological application of pyrolysis oil lies on the carbohydrate components, in particular levoglucosan. Levoglucosan was either converted to glucose or used directly for biotechnological processes, e.g., ethanol production and itaconic acid production (Nakagawa et al., 1984; Luque et al., 2014). *Aspergillus niger* CX-209 was cultivated for citric acid production both, on pure levoglucosan and on a cotton based pyrolysis oil (Zhuang et al., 2001). Using pure cellulose as a feedstock results in a levoglucosan rich pyrolysis oil containing low amounts of other organic and lignin-derived compounds making a comparison to the pyrolysis oil used in this study impossible. Tests with similar crude pyrolysis oil are rare. Yang et al. (2011) analyzed the growth of six fungi based on either pure pyrolysis oil or pyrolysis oil added to potato medium in concentration between 0 and 0.3%. Two of the tested fungal species, *Aspergillus niger* and *Phanerochaete chrysosporium*, were also tested in this study and found to tolerated up to 0.5 and 1 % pyrolysis oil, respectively (compare **Table 1**). We tested as the first fungi of all phyla and showed that most fungi are able to tolerate much higher concentrations of pyrolysis oil than tested before. Furthermore the high tolerance level of *T. versicolor* and the ability of growth with up to 1% pyrolysis oil of *A. oryzae* makes crude pyrolysis oil to a possible carbon source for biomass formation, but not for organic acid production. The tolerance of higher amounts of pyrolysis oil of *T. versicolor* is attributed to the fact that these organism is a lignin degrader and can probably handle aromatic compounds in the oil. However, the results also give an insight into the complex toxic effects of the different compounds within pyrolysis oil. The 11 chosen substances were analyzed as representatives of the different chemical groups, like organic acids, phenolic compounds, and lactones. Because glucose was used as carbon source in all tolerance experiments the main focus of this work was to discover and describe the toxicity of the chemical compounds in pyrolysis oil and the reason for the observed production and growth limits. These results are also important for biotechnological application of other pretreated biomass containing similar substances as contaminants. Additionally, some of the analyzed chemicals are also relevant as environmental pollutants, and therefore this work could be helpful in the field of fungal bioremediation.

Following, the effects of the single substances and possible inhibition mechanisms are discussed. With regard to organic acids, the results show that addition of propionic acid has a major influence on the growth of *A. oryzae* and *R. delemar*. Even in the presence of low propionic acid concentration, i.e., 0.1%, growth was only observed for *R. delemar*. This observation is consistent with numerous studies that report a growth inhibition of *A. flavus*, a close relative to *A. oryzae*, with increasing concentration of propionic acid (Ghosh and Häggblom, 1985). Interestingly, propionic acid had a minor influence on malic acid production as 33.66 ± 2.47 g/L malic acid was produced in the presence of 1.3% propionic acid concentration, which corresponds to its content in pyrolysis oil. This is also valid for the yields. Propionic acid has a higher impact to the fumaric acid production with *R. delemar*, where a rising concentration leads to a lower production in total but does not influence the yields, being the highest at 1.3% propionic acid. For every other concentration the yields are lower than in the control. However, acetic acid, which is the main component of pyrolysis oil with approximately 5%, didn’t show any toxic effects, and in contrast, it can be used as carbon source for *A. oryzae* (Oswald et al., 2016).

With respect to small molecular compounds, the addition of ethylene glycol had only a minor influence on growth. A slight decrease in the formed malate concentrations and the colony diameter with increasing concentrations of ethylene glycol was observed. Regarding malic acid production, ethylene glycol had also only minimal effects to the yields. Alcohol oxidase, which was discovered and described in *A. ochraceus* (Isobe et al., 2007), and is responsible for the degradation of ethylene glycol, might be accountable for the observed tolerance toward ethylene glycol in this study. However, a decrease of ethylene glycol during cultivation time could not be verified.

One of the most problematic substances tested is 2-cyclopenten-1-one, with which growth and malic acid production are only possible at very low concentrations. The presence of this substance had a greater influence on the growth of *A. oryzae* than on malic acid production, but in general it is toxic for both fungi in very low concentrations. In contrast to *A. oryzae*, it leads with *R. delemar* also to a very low yield of 0.1 g/g. Due to the low inhibitory concentration, a hormonal effect of 2-cyclopenten-1-one might be conceivable. In cell culture it could be shown that 2-cyclopenten-1-one induces the production of the heat shock protein 70 (HSP 70) in human cells, and interferes with protein expression (Rossi et al., 1996). Another possible reason for the high inhibitory potential of 2-cyclopenten-1-one might be the inhibition of important metabolic pathways, which are not yet described for this substance. Similarly, when adding furfural to agar plates, growth was strongly inhibited for all tested concentrations. Even at a furfural concentration of 0.05% fungal growth was inhibited. The growth inhibition could, as known for *E. coli*, be caused by a low availability of sulfur-containing amino acids. The inhibition of the synthesis of these amino acids could be observed in presence of furfural (Miller et al., 2009). Another possible reason for a growth inhibition is the chemical reactivity of the aldehyde furfural. This reactivity has been suggested to be the reason for the toxicity or furfural (Zaldivar et al., 1999). However, the influence of furfural on malic acid production was less pronounced. In contrast, hydroxyacetone was tolerated in very high concentration compared to other tested substances for both fungi. Because of the solvent properties of hydroxyacetone, a change in the ambient conditions due to hydroxyacetone addition might possibly be leading to increased cell membrane disorganization, especially in the higher concentration ranges, resulting in an inhibition of growth and malic acid production. Surprisingly, the impact of the product yield was considerably higher for *R. delemar* with a yield of 0.15 g/g, in contrast to minor effect on malic acid production with *A. oryzae*.

Although γ-butyrolactone is also used as a solvent, it shows apparently no effects to the cells at the tested concentrations. Yet, in the presence of all tested γ-butyrolactone concentrations inhibition of growth and malic acid production of *A. oryzae* was not observed. Furthermore, it might be possible that *A. oryzae* is able to metabolize this lactone as a carbon source. The ability to degrade THF is described for some Ascomycota like *Aureobasidium pullulans* (Patt and Abebe, 1995). This could be an indication for possible degradation by *A. oryzae*. However, degradation of γ-butyrolactone was not observed by the used analysis methods and the slightly lower yield for malic acid would present a disqualification for its possible usage as carbon source.

The addition of the phenolic compounds phenol, *o*-, *m*-, *p*-cresol, guaiacol, syringol, and isoeugenol, resulted in a strong inhibition of growth even at low concentrations. Phenol is a well-known biocide, *o*-, *m*-, *p*-cresol is frequently used as fungicide. Its toxicity is based on its membrane activity (Mörsen and Rehm, 1990). Similar effects of guaiacol on *A. parasiticus* have been published (Pillai and Ramaswamy, 2012). At the lowest tested concentration of phenol, cresols, and guaiacol, more malic acid was detected than in the control approach, indicating either a promoting effect of phenol on malic acid production or a better release of the organic acids from the cell. The possibility of an enhanced release of malate and fumarate in the culture medium could be explained by a potentially occurring permeabilization of the cell membrane by the presence of phenol. It is known that one bottleneck of the malic acid production is the export from cytoplasm to medium (Brown et al., 2013) and therefore the permeabilization of the membrane could lead to an accelerated transport by the concentration gradient. With increasing concentrations of phenol, malic acid production drops off quickly due to the toxic effects of increasing cell membrane permeabilization. Compared to that, the yields of malic acid rises with an increase of phenol except the lowest phenol concentration where the yield is extremely high (0.86 g/g) which supports the permeabilization theory.

Although it is known that *A. oryzae* has genes encoding for enzymes enabling the degradation of *m*-cresol (Machida et al., 2005), it seems that *A. oryzae* only tolerates cresol in very low concentrations (growth limit 0.05%; production limit 0.03%). In a study dealing with the degradation of phenol, *p*-cresol, *m*-cresol, and *o*-cresol by various fungal species it was shown that *Aspergillus* sp. is less capable of degrading phenol and cresol compared to other fungi (Atagana, 2004). The reason for the very sharp transition from less influence to total inhibition, which leads to the classification of cresol to the first group in contrast to phenol, could be that a mixture of *o*-, *m*-, and *p*-cresol was tested. Contrary to the other substances, the cresol mixture shows lower toxicity limits to malic acid production than to growth.

Even at low concentrations of syringol inhibition of growth and malic acid with *A. oryzae* production was observed with a correlation between decreasing malic acid production and increasing concentrations of syringol. During growth tests on agar plates, as well as during production in liquid culture medium, decolorization of former violet medium was observed indicating for a possible degradation or derivatization of syringol. The yield of malic acid increased from 0.04 g/g with 0.3% syringol to 0.2 g/g with 0.17% guaiacol, which supports the hypothesis of degradation. However, this kind of degradation or derivatization seems to happen simultaneously to growth or production and does not result in a delay of malic acid production as observed for isoeugenol. The antifungal effect of isoeugenol to the surface growth of various *Aspergillus* species was already described in 1996. In these studies, strong inhibition of growth was observed on agar plates with 0.02% isoeugenol and finally a total inhibition at the same concentration as in this study (0.03%) (Mansour et al., 1996). Regarding the malic acid production profile in the presence of isoeugenol, it is fundamentally different to the production curves of other analyzed substances. The start of the malic acid production is delayed: the higher the concentration of isoeugenol, the later the production started, until no production was observed during cultivation time. A possible reason for this phenomenon could be the degradation or derivatization of isoeugenol until a more tolerated concentration is reached. This process needs energy, which is produced during cell respiration, so as long as energy is needed for the derivatization of the toxic substance, malic acid production does not take place. This theory is supported by consideration of the malic acid yields: In the presence of 0.6% isoeugenol, the yield of fumaric acid with *R. delemar* is the lowest measured. In general, fungal mycelium appears to tolerate higher amounts of toxic substances if it does not grow as during the organic acid production phase. Because of the novelty of this observation, the reasons can only be speculated. During the production phase, the fungal growth is limited by nitrogen deficiency, leading to a decrease of metabolic activity and a shift of resources to acid production. This might bring a lower susceptibility to toxic effects.

## Conclusion

In summary, it was observed that *A. oryzae* and *R. delemar* tend to be more tolerant toward toxic compounds during the acid production phase, when less biomass is formed than during the active growth phase. Therefore, using pyrolysis oil or fractions thereof for fermentation processes might be possible when the growth phase and the production phase are separated and production takes place during stationary growth. Although this applies to the individual substances *A. oryzae* tolerates higher concentrations of pyrolysis oil during growth phase (2%) than during malic acid production phase (1%) during the observed cultivation time. One compound was identified to be a main reason for the low tolerance level of pyrolysis oil: 2-cyclopenten-1-one, which is present in a concentration of 0.308%. In growth tolerance tests with *A. oryzae*, the growth limit of this substance was observed at a concentration of 0.00625% corresponding to approximately 2% of the content in pyrolysis oil. This is consistent to the growth limit with pyrolysis oil (2%). In tolerance tests the growth and also fumaric acid production limit of *R. delemar* is even lower at 0.005%, which corresponds to approximately 1.6%. However, for malic acid production, the limit was at a concentration of 0.0125% corresponding to approximately 4% of the content in pyrolysis oil. Therefore, this substance alone is not responsible for the acid production limit of pyrolysis oil (1%). The fumaric acid production limit in presence of isoeugenol (0.005%) corresponds with to approximately 1% pyrolysis oil and for growth the limit is near 5%. Possible synergistic effects of the analyzed and also not analyzed substances could not be tested because of the large number of possible combinations and low availability of chemicals. Strong synergistic effects of furfural in combination with other aldehydes were described in former studies (Zaldivar et al., 1999). Moreover, chemical reactions between the components also be possible, which would lead to new unknown substances. However, even by testing single substances, the results give an idea of the complex nature of pyrolysis oil with many possible and different inhibition mechanisms of its compounds.

## Author Contributions

SD: Substantial performance of the experiments and data evaluation. Writing the manuscript as first author. JK: Substantial performance of the experiments with *A. oryzae*. MB: Substantial performance of the experiments with *R. delemar*. ND: Substantial contribution and production of the pyrolysis oil. Writing the part of pyrolysis oil production. CS: Substantial contribution to manuscript concept and advisory. Critically revising of the final manuscript version. KO: Substantial contribution to the conception of the experiments and advisory of the lab work. Critically revising of the final manuscript version.

## Conflict of Interest Statement

The authors declare that the research was conducted in the absence of any commercial or financial relationships that could be construed as a potential conflict of interest.
