# Supplementary material for: Evaluation of Pyrolysis Oil as Carbon Source for Fungal Fermentation
Source: Front Microbiol. 2016 Dec 22;7:2059. doi: 10.3389/fmicb.2016.02059 (PMC5177650; doi:10.3389/fmicb.2016.02059)
Supplement: Supplementary file 1 [file Table_1.pdf]

Composition of pyrolysis oil, classified by kind of substance and concentration.

CAS-No.	Compound	wt. %
**Non-aromatic compounds**		
**64-19-7**	Acetic acid	5.004
**79-09-4**	Propionic acid	1.302
**107-92-6**	Butyric acid	0.164
**-----**	poss. Propanoid acid, ethenyl ester	
**107-21-1**	Ethylene glycol	1.258
**141-46-8**	Hydroxyacetaldehyde	0.314
**116-09-6**	Hydroxypropanone (Acetol)	4.631
**110-13-4**	2,5-Hexandione (Acetonylacetone)	0.039
**78-93-3**	2-Butanone	0.038
**5077-67-8**	1-Hydroxy-2-Butanone	0.844
**431-03-8**	2,3-Butandione (Diacetyl)	0.036
**513-86-0**	3-Hydroxy-2-Butanone (Acetoin)	0.119
**592-20-1**	1-Acetyloxy-Propan-2-one	0.2018
**120-92-3**	Cyclopentanone	0.095
**930-30-3**	2-Cyclopenten-1-one	0.308
**1121-05-7**	2,3-Dimethyl-2-Cyclopenten-1-one	0.222
**1120-73-6**	2-Methyl-2-Cyclopenten-1-one	0.117
**2758-18-1**	3-Methyl-2-Cyclopenten-1-one	0.229
**566-26-99**	3-Ethyl-2-Cyclopenten-1-one	0.056
**10493-98-8**	2-Hydroxy-2-Cyclopenten-1-one	0.032
**80-71-7**	2-Hydroxy-1-methyl-1-Cyclopenten-1-one	0.939
**21835-01-8**	3-Ethyl-2-hydroxy-2-Cyclopenten-1-one	0.300
**930-68-7**	2-Cyclohexen-1-one	0.024
	poss: 2-Butenone	0.013
	Isomer of 3-Methyl-2-Cyclopenten-1-one	0.019
	Isomer of 3,4-Dimethyl-Cyclopentenone	0.034
	Dimethyl-Cyclopenten-one	0.026
	poss: 1-Acetyloxy-Butan-2-one	0.027
	Isomere of 2,3-Dimethyl-2-Cyclopenten-1-one	0.062
	Derivative of Dimethyl-Cyclopentenone	0.060
	2,3,4-Trimethyl-2-Cyclopenten-1-one	0.068
	Isomer of 3-Ethyl-2-hydroxy-Cyclopenten-1-one	0.094
	poss: 2-Cyclohexene-1,1-dione	0.028
	poss: 2,4-dimethyl-1,3-Cyclopenten-1-one	0.049
	Trimethyl-2-Cyclopenten-1-one	0.029
	poss: Trimethyl-2-Cyclopenten-1-one	0.035
	poss: 2-hydroxy-3-propyl-2-c-Cyclopenten-1-one	0.036
	2-Heptadecanone	0.108
	poss: 2-Pentadecanone or Isomere	0.029

**Heterocyclic Compounds**		
**98-00-0**	2-Furfuryl alcohol	0.124
**2082-571-2**	2(3H)-Furanone	0.065
**497-23-4**	2(5H)-Furanone	0.176
**98-01-1**	2-Furaldehyde	0.265
**498-60-2**	3-Furaldehyde	0.026
**620-02-0**	5-Methyl-2-Furaldehyd	0.032
**1192-62-7**	1-(2-Furanyl)-Ethanone	0.041
**22122-36-7**	(5H)-3-Methyl-Furan-2-one	0.094
	2,5-Dehydro-3,5-Dimethyl-Furan-2-one	0.127
**96-48-0**	γ-Butyrolactone	0.335
	poss: 5-Methyl-2(5H)-Furanone	0.042
	4-Methyl-(5H)-Furan-2-one	0.081
	poss: Isomere of 2,5-Dihydro-3,5-dimethyl-Furan-2-one	0.044
	Lactone derivative	0.037
**Aromatic Compounds**		
**4265-25-2**	2-Methyl-Benzofuran	0.016
**83-33-0**	2,3-Dihydro-1H-Inden-1-one	0.44
	Benzene	0.005
	2H-1-3,4-Dihydro-6-hydroxy-Benopyran-2-one	0.012
	poss: 3-Hydroxy-Benzaldehyde	0.048
**98-86-2**	Acetophenone	0.017
**108-95-2**	Phenol	0.384
**95-48-7**	o-Cresol	0.151
**106-44-5**	p-Cresol	0.134
**108-39-4**	m-Cresol	0.170
**95-87-4**	2,5-Dimethyl-Phenol	0.068
**105-67-9**	2,4-Dimethyl-Phenol	0.053
**576-26-1**	2,6-Dimethyl-Phenol	0.047
**526-75-0**	2,3-Dimethyl-Phenol	0.03
**108-68-9**	3,5-Dimethyl-Phenol	0.03
**527-60-6**	2,4,6-Timethyl-Phenol	0.012
**90-00-6**	2-Ethyl-Phenol	0.065
**620-17-7**	3-Ethyl-Phenol	0.067
**123-07-9**	4-Ethyl-Phenol	0.171
	4-Vinyl-Phenol	0.308
**401-92-8**	Trans-4-propenyl-Phenol	0.061
	Derivative of 2,3,4- or 2,4,5-Trimethyl-Phenol	0.034
	Ethyl-Methyl-Phenol	0.040
	Trimethyl-Phenol	0.030
**90-05-1**	Guaiacol	0.469
**93-51-6**	4-Methyl-Guaiacol	0.150
**2785-89-9**	4-Ethyl-Guaiacol	0.191
**7786-61-0**	4-Vinyl-Guaiacol	0.412
**97-53-0**	4-Allyl-Guaiacol (Eugenol)	0.085
**2785-87-7**	4-Propyl-Guaiacol	0.039

**97-54-1**	Cis-4-Propenyl-Guaiacol (Isoeugenol)	0.144
**5932-68-3**	Trans-4-Propenyl-Guaiacol (Isoeugenol)	0.524
**121-33-5**	Vanillin	0.200
**498-02-2**	4-Hydroxy-3-methoxy-Phenylethanone (Acetoguajacone)	0.099
**2503-46-0**	Guaiacylacetone	0.094
**458-36-6**	Coniferylaldehyde	0.023
**91-10-1**	Syringol	0.556
	4-Methyl-Syringol	0.162
	4-Ethyl-Syringol	0.094
	4-Vinyl-Syringol	0.218
**6627-88-9**	4-Allyl-Syringol	0.079
	4-Propyl-Syringol	0.048
**627-88-9**	Cis-4-(1-propenyl)-Syringol	0.077
	Trans-4-(1-propenyl)-Syringol	0.299
**134-96-3**	Syringaldehyde	0.177
**2478-38-8**	Acetosyringone	0.095
	Propiosyringone	0.059
	Syringylacetone	0.042
	**Carbohydrates**	
**7732-18-5**	1,5-Anhydro-β-D-arabinofuranose	0.238
**498-07-7**	1,6-Anhydro-β-D-glucopyranose (Levoglucosan)	0.965
	1,4:3,6-Dianhydro-α-D-glucopyranose	0.431
	1,2-Ethanediol-monoacetate	0.048
	unknown compunds	0.251
